# Supplementary material for: The potential of the transformer-based survival analysis model, SurvTrace, for predicting recurrent cardiovascular events and stratifying high-risk patients with ischemic heart disease
Source: PLoS One. 2024 Jun 18;19(6):e0304423. doi: 10.1371/journal.pone.0304423 (PMC11185454; doi:10.1371/journal.pone.0304423)
Supplement: S1 File — This file contains the code to execute the multiple imputation method in Python. (DOCX) [file pone.0304423.s001.docx]

# In[1]:

import os

import pandas as pd

import numpy as np

# In[2]:

df =pd.read_csv(your_data_path)

# In[3]:

from sklearn.model_selection import train_test_split

train, test = train_test_split(df, test_size=0.1, random_state=0)

print(train.shape)

print(test.shape)

# In[4]:

np.random.seed(0)

state_num = np.random.randint(0, 10000, 5)

print(state_num)

# In[5]:

from sklearn.experimental import enable_iterative_imputer

from sklearn.impute import IterativeImputer

for i in state_num:

imp = IterativeImputer(max_iter= 500, random_state=i, sample_posterior=True)

train_imp = imp.fit_transform(train)

train_imp = pd.DataFrame(train_imp, columns=df.columns)

train_imp.to_csv(Your_train_data_path)

test_imp = imp.transform(test)

test_imp = pd.DataFrame(test_imp, columns=df.columns)

test_imp.to_csv(Your_test_data_path)
