# Supplementary material for: The potential of the transformer-based survival analysis model, SurvTrace, for predicting recurrent cardiovascular events and stratifying high-risk patients with ischemic heart disease
Source: PLoS One. 2024 Jun 18;19(6):e0304423. doi: 10.1371/journal.pone.0304423 (PMC11185454; doi:10.1371/journal.pone.0304423)
Supplement: S2 File — This file contains the code to execute SurvTrace in Python. (DOCX) [file pone.0304423.s002.docx]

# In[1]:

import pandas as pd

import numpy as np

from sklearn.model_selection import train_test_split

from sklearn.preprocessing import KBinsDiscretizer, LabelEncoder, StandardScaler

import pdb

from SurvTRACE.survtrace.utils import LabelTransform

from SurvTRACE.survtrace.evaluate_utils import Evaluator

from SurvTRACE.survtrace.utils import set_random_seed, pad_col

from SurvTRACE.survtrace.model import SurvTraceSingle, SurvTraceMulti

from SurvTRACE.survtrace.train_utils import Trainer

from SurvTRACE.survtrace.config import STConfig

import matplotlib.pyplot as plt

import math

import warnings

warnings.filterwarnings('ignore')

import xfeat

import shap

import torch

shap.initjs()

# In[2]:

csv_path = 'Your DatafilePath'

categorical_cols = [cat1, cat2, cat3]

numerical_cols = [num1, num2, num3]

target_col = target_outcome

time_col = target_outcome_time

horizons = [0.25, 0.5, 0.75]

# In[3]:

df = pd.read_csv(csv_path)

df = pd.concat([xfeat.SelectCategorical().fit_transform(df[categorical_cols].astype('object')),

xfeat.SelectNumerical().fit_transform(df[numerical_cols]) , df.iloc[:, -2:]], axis = 1)

df = df.rename(columns={target_col: 'event', time_col: 'duration'})

df = df.astype({'duration':'int32'})

times_ = np.quantile(df["duration"][df["event"]==1.0], horizons).tolist()

observe_d = np.array([df["duration"].min()]+times_+[df["duration"].max()])

labtrans = LabelTransform(cuts=observe_d)

STConfig['horizons'] = horizons

STConfig['labtrans'] = LabelTransform(cuts=observe_d)

STConfig['num_numerical_feature'] = int(len(numerical_cols))

STConfig['num_categorical_feature'] = int(len(categorical_cols))

STConfig['num_feature'] = int(len(df.columns[:-2]))

STConfig['duration_index'] = labtrans.cuts

STConfig['out_feature'] = int(labtrans.out_features)

#Change according to your data.

STConfig['early_stop_patience'] = 10

STConfig['num_attention_heads'] = 2

STConfig['num_hidden_layers'] = 4

STConfig['hidden_size'] = 4

STConfig['intermediate_size'] = 4

STConfig['initializer_range'] = 1

STConfig['attention_probs_dropout_prob'] = 0.2

# In[4]:

def dataloader(df, random_state_num=42):

max_duration_idx = df["duration"].argmax()

df_max = df.iloc[max_duration_idx:max_duration_idx + 1]

df2 = df.drop(max_duration_idx)

df_Train, df_val = train_test_split(df2, test_size=0.25, random_state=random_state_num)

df_Trn = pd.concat([df_max, df_Train], axis = 0)

return df_Trn, df_val

# In[5]:

def df_labtrans_trn(df):

get_target = lambda df: (df['duration'].values, df['event'].values)

_cols_categorical = categorical_cols

_cols_standardize = numerical_cols

_duration = observe_d

df_feat = df.drop(["duration","event"],axis=1)

df_feat = pd.concat([df_feat[_cols_categorical], df_feat[numerical_cols]], axis=1)

_vocab_size = 0

for _,feat in enumerate(_cols_categorical):

df_feat[feat] = LabelEncoder().fit_transform(df_feat[feat]).astype(float) + _vocab_size

_vocab_size = df_feat[feat].max() + 1

_labtrans = LabelTransform(cuts=_duration)

_labtrans.fit(*get_target(df))

y = _labtrans.transform(*get_target(df)) # y = (discrete duration, event indicator)

df_y = pd.DataFrame({"duration": y[0], "event": y[1], "proportion": y[2]}, index=df.index)

return df_feat, df_y, _vocab_size

# In[6]:

from sksurv.metrics import concordance_index_censored

import copy

def Cindex(df_y, risk, quantile = horizons):

get_target = lambda df: (df['duration'].values, df['event'].values)

durations_test, events_test = get_target(df_y)

et_test = np.array([(events_test[i], durations_test[i]) for i in range(len(events_test))],

dtype = [('e', bool), ('t', float)])

_risk = risk

_horizons = copy.deepcopy(quantile)

_horizons.insert(0, 0.0)

_horizons.append(1.0)

print('------')

for i in range(len(horizons) + 2):

ci =concordance_index_censored(et_test['e'], et_test['t'], _risk[:, i])

print(f"For {_horizons[i]} quantile,")

print("Concordance_index:", ci[0])

print('------')

return ci[0]

# In[7]:

train, valid = dataloader(df)

# In[8]:

def objective(trial):

STConfig['num_hidden_layers'] = trial.suggest_int('num_hidden_layers', 16, 128, step = 4)

STConfig['hidden_size'] = trial.suggest_int('hidden_size', 16, 256, step = 16)

STConfig['intermediate_size'] = trial.suggest_int('intermediate_size', 16, 128, step = 4)

STConfig['num_attention_heads'] = trial.suggest_categorical('num_attention_heads', [2, 4, 8, 16])

STConfig['initializer_range'] = trial.suggest_uniform("initializer_range", 0, 0.1)

STConfig['attention_probs_dropout_prob'] = trial.suggest_uniform('attention_probs_dropout_prob', 0.1, 0.5)

hparams = {

'batch_size': trial.suggest_int('train_batch_size', 16, 128),

'weight_decay': 0,

'learning_rate': trial.suggest_loguniform('learning_rate', 1e-8, 0.001),

'epochs': 50,

'val_batch_size': trial.suggest_int('val_batch_size', 8, 64),

}

X_train, y_train, vocab_size = df_labtrans_trn(train)

X_valid, y_valid, _ = df_labtrans_trn(valid)

STConfig['vocab_size'] = int(vocab_size)

model = SurvTraceSingle(STConfig)

trainer = Trainer(model)

train_loss_list, val_loss_list = trainer.fit(

(X_train, y_train), (X_valid, y_valid),

**hparams

)

evaluator = Evaluator(df, X_train.index)

val_results = evaluator.eval(model, (X_valid, valid.iloc[:, -2:]))

val_risks = model.predict_risk(X_valid)

return Cindex(valid.iloc[:, -2:], val_risks.to('cpu'))

# In[9]:

import optuna

study_name = hoge # Unique identifier of the study.

study = optuna.create_study(study_name=study_name,

storage= hogehoge,

load_if_exists=True,

direction='maximize')

study.optimize(objective,

n_trials=20

)

# In[10]:

print(study.best_value)

print(study.best_params)

# In[11]:

STConfig['num_hidden_layers'] = study.best_params['num_hidden_layers']

STConfig['hidden_size'] = study.best_params['hidden_size']

STConfig['intermediate_size'] = study.best_params['intermediate_size']

STConfig['initializer_range'] = study.best_params['initializer_range']

STConfig['num_attention_heads'] = study.best_params['num_attention_heads']

STConfig['attention_probs_dropout_prob'] = study.best_params['attention_probs_dropout_prob']

hparams = {

'batch_size': study.best_params['train_batch_size'],

'weight_decay': 0,

'learning_rate': study.best_params['learning_rate'],

'epochs': 50,

'val_batch_size':study.best_params['val_batch_size']

}

# In[12]:

X_train, y_train, vocab_size = df_labtrans_trn(train)

X_valid, y_valid, _ = df_labtrans_trn(valid)

X_test, y_test, __ = df_labtrans_trn(df_Test_)

STConfig['vocab_size'] = int(vocab_size)

model = SurvTraceSingle(STConfig)

trainer = Trainer(model)

train_loss_list, val_loss_list = trainer.fit(

(X_train, y_train), (X_valid, y_valid),

**hparams

)

#evaluate model

evaluator = Evaluator(df, X_train.index)

val_results = evaluator.eval(model, (X_valid, valid.iloc[:, -2:]))

val_risks = model.predict_risk(X_valid)

test_results = evaluator.eval(model, (X_test, df_Test_.iloc[:, -2:]))

test_risks = model.predict_risk(X_test)

# In[13]:

Cindex(valid.iloc[:, -2:], val_risks.to('cpu'))

# In[14]:

Cindex(df_Test_.iloc[:, -2:], test_risks.to('cpu'))
