# Supplementary material for: The potential of the transformer-based survival analysis model, SurvTrace, for predicting recurrent cardiovascular events and stratifying high-risk patients with ischemic heart disease
Source: PLoS One. 2024 Jun 18;19(6):e0304423. doi: 10.1371/journal.pone.0304423 (PMC11185454; doi:10.1371/journal.pone.0304423)
Supplement: Attachment — Submitted filename: Respons_To_Reviewers_20240424.docx [file pone.0304423.s004.docx]

**Reviewer #1: Thank you for addressing my concerns.**

**The outcome occurs in 8% of the population.**

**How would you manage this imbalance in outcomes?**

Thank you for your valuable comments regarding the imbalanced data on outcomes. We appreciate your input, as the issue of imbalanced data is one of the critical challenges in developing machine learning models.

In this study, we did not employ oversampling or undersampling methods to address the imbalanced data for the following reasons:

Undersampling method

The undersampling method reduces the number of majority class instances to match the level of minority class instances, resulting in a decrease in the overall sample size. SurvTRACE, the analysis method used in this study, is derived from the Transformer and requires a larger sample size. We were concerned that a reduction in sample size might impact the performance of SurvTRACE; therefore, we decided against adopting the undersampling method.

Oversampling method and SMOTE

The oversampling method and SMOTE generate synthetic data by referring to minority class instances. In this study, we used a multiple imputation method to handle missing values and generate pseudo-complete data for analysis. We were concerned that creating additional synthetic data based on the already imputed data might result in a noisier dataset. Consequently, we decided against adopting these methods. Moreover, the report by Wallace et al. (2011 IEEE 11th International Conference on Data Mining, Vancouver, BC, Canada, 2011, pp. 754-763, doi: 10.1109/ICDM.2011.33.) indicates that applying SMOTE to imbalanced datasets with an outcome incidence rate of 5-10% and a dimensionality exceeding 100 may not lead to significant improvements in accuracy. The outcome incidence rate in our study is 8%, and the dimensionality is 171, which falls within the range indicated in Wallace et al.'s report.

Based on the above reasons, we decided not to adopt oversampling or undersampling methods for the imbalanced data in this study and instead performed the analysis using the original dataset as is.

However, as you have rightly pointed out, addressing the imbalanced data problem is crucial for improving the performance of machine learning models. In future research, we will strive to explore more effective methods for dealing with imbalanced data and aim to enhance the accuracy of the models.

We sincerely appreciate your valuable comments, which we believe will contribute to the improvement of our research.

**Reviewer #2: The authors have addressed all my concerns. I just have two suggestions.**

**1. The authors should consider removing the word "retrospectively" and replacing "post-index" with "post-index event" in the following sentence (in the abstract):**

**"Each patient’s initial PCI at our hospital was designated as the index procedure, and a composite of major adverse cardiovascular events (MACE) was retrospectively monitored for up to two years post-index."**

**2. The authors should also consider replacing "selective bias" with "selection bias."**

We greatly appreciate your valuable suggestions for improving our manuscript. We have carefully considered your comments and made the following changes:

Regarding your first suggestion, we agree that removing the word "retrospectively" and replacing "post-index" with "post-index event" will enhance the clarity of the sentence. We have revised the sentence in the abstract as follows:

Original (Page 1, Lines 12-14): "Each patient's initial PCI at our hospital was designated as the index procedure, and a composite of major adverse cardiovascular events (MACE) was **retrospectively** monitored for up to two years **post-index.**"

Revised (Page 1, Lines 12-14): "Each patient's initial PCI at our hospital was designated as the index procedure, and a composite of major adverse cardiovascular events (MACE) was monitored for up to two years **post-index event.**"

As per your second suggestion, we have replaced the term "selective bias" with "selection bias" throughout the manuscript to ensure the correct terminology is used.

Original (Page 19, Lines 302-304): "Despite this thorough tracking, some events might have been overlooked as a result of patients relocating or transferring to other hospitals, potentially leading to **selective bias.**"

Revised (Page 19, Lines 302-304): "Despite this thorough tracking, some events might have been overlooked as a result of patients relocating or transferring to other hospitals, potentially leading to **selection bias.**"

We believe that these changes, based on your insightful comments, will significantly improve the quality and readability of our manuscript. We sincerely appreciate the time and effort you have taken to review our work and provide such constructive comments.

Thank you once again for your valuable suggestions.
